# Letter to Editor on “Association between gallstone disease and carotid intima-media thickness: a prospective observational cross-sectional study in a tertiary care center”

**DOI:** 10.1097/MS9.0000000000003134

**Published:** 2025-03-07

**Authors:** Uzair Shahid, Alishba Rauf Ahmed, Farah Aziz Sawal, Malik Olatunde Oduoye

**Affiliations:** a Watim Medical College, University of Health Sciences, Lahore, Pakistan; b Khyber Medical University – Institute of Medical Sciences, Kohat, Pakistan; c University Medical and Dental College Faisalabad, Faisalabad, Pakistan; d The Medical Research Circle, Bukavu, Democratic Republic of Congo

We would like to commend Thapa *et al*^[1]^ for their insightful study titled “Association between Gallstone Disease and Carotid Intima-Media Thickness: A Prospective Observational Cross-Sectional Study in a Tertiary Care Center.” Their research significantly enhances our understanding of the relationship between gallstone disease (GD) and increased cardiovascular risk, as indicated by carotid intima-media thickness (CIMT). This study is an important step in recognizing the broader cardiovascular implications of GD, highlighting the need for thorough cardiovascular risk assessments in patients diagnosed with this condition.

However, we would like to point out a few areas where the study could be further strengthened. The study’s sample size of 96 participants, while informative, is relatively small compared to other studies, such as Kim *et al*^[2]^, which included 330 participants. A larger cohort could have improved the statistical power and the generalizability of the results.

Additionally, the study relied solely on ultrasonography to measure CIMT, which, while effective, might have benefited from the inclusion of other imaging modalities. For example, Serin et al.^[3]^ employed both ultrasonography and CT scans to provide a more comprehensive vascular assessment. Incorporating multiple imaging techniques could have allowed for a more detailed evaluation of vascular health, potentially leading to stronger conclusions.

Furthermore, while the study primarily focused on CIMT as the only marker of cardiovascular risk, Serin *et al*^[3]^ also examined additional vascular parameters, such as plaque formation and calcification, which offer a more complete picture of cardiovascular risk. Expanding the scope of vascular assessment in Thapa *et al*’s study could have provided deeper insights into the relationship between GD and cardiovascular disease.

Moreover, while Thapa *et al*^[1]^ accounted for basic demographic variables such as age, sex, and BMI, the exclusion criteria could have been more stringent to minimize potential confounding factors. For instance, Serin *et al* excluded patients with significant comorbid conditions like coronary artery disease, which helps reduce confounding and increases the reliability of the findings.

The study could also benefit from a more detailed gender-specific analysis. Kim *et al*^[2]^ focused on male participants and provided an in-depth exploration of gender-related differences in cardiovascular risk. A similar approach in Thapa *et al*’s study might uncover important gender-specific patterns that could enhance our understanding of the cardiovascular implications of GD.

Additionally, an important aspect discussed by Yu *et al*,^[4]^ but not addressed by Thapa *et al*, is the use of brachial-ankle pulse wave velocity (baPWV) as a measure of arterial stiffness progression in patients with GD. Yu *et al* highlighted baPWV as a significant non-invasive marker for assessing arterial stiffness, identifying GD as an independent predictor of increased baPWV. Including such measures could offer a more comprehensive understanding of the subclinical atherosclerosis process in GD patients, further improving the predictive value of cardiovascular assessments.

Lastly, while the statistical methods employed by Thapa *et al* were appropriate, they were relatively basic. In contrast, Kim *et al*^[2]^ used advanced regression analysis to identify independent predictors of cardiovascular risk. Applying more sophisticated statistical techniques could refine the study’s conclusions and provide a clearer understanding of the relationship between GD and cardiovascular risk.

In conclusion, while Thapa *et al*’s study provides valuable insights into the association between gallstone disease and cardiovascular risk, it could be further strengthened by including a larger sample size, utilizing multiple imaging modalities, expanding the scope of vascular assessment, applying more stringent exclusion criteria, conducting detailed gender-specific analysis, incorporating baPWV measurement, and employing advanced statistical methods. These enhancements would increase confidence in the findings and contribute more comprehensively to our understanding of the cardiovascular risks associated with gallstone disease.

## Data Availability

Not applicable.
